# A degradation fragment of type X collagen is a real-time marker for bone growth velocity

**DOI:** 10.1126/scitranslmed.aan4669

**Published:** 2017-12-06

**Authors:** Ryan F. Coghlan, Jon A. Oberdorf, Susan Sienko, Michael D. Aiona, Bruce A. Boston, Kara J. Connelly, Chelsea Bahney, Jeremie LaRouche, Sarah M. Almubarak, Daniel T. Coleman, Irute Girkontaite, Klaus von der Mark, Gregory P. Lunstrum, William A. Horton

**Affiliations:** 1 Research Center, Shriners Hospitals for Children, Portland, OR 97239, USA; 2 Department of Molecular and Medical Genetics, Oregon Health and Science University, Portland, OR 97239, USA; 3 Department of Pediatrics, Oregon Health and Science University, Portland, OR 97239, USA; 4 Department of Orthopedic Surgery, University of California Medical School, San Francisco, CA 94111, USA; 5 Graduate School of Social Service, Fordham University, Lincoln Center, New York, NY 10023, USA; 6 Department of Immunology, State Research Institute, Vilnius University, Vilnius, Lithuania; 7 Freidrich-Alexander University, Erlangen, Germany

## Abstract

Despite its importance as a key parameter of child health and development, growth velocity is difficult to determine in real timebecause skeletal growth is slowand clinical tools to accurately detect very small increments of growth do not exist. We report discovery of a marker for skeletal growth in infants and children. The intact trimeric noncollagenous 1 (NC1) domain of type X collagen, the markerwe designated as CXMfor Collagen X Marker, is a degradation by-product of endochondral ossification that is released into the circulation in proportion to overall growth plate activity. Thismarker corresponds to the rate of linear bone growth at timeofmeasurement. Serumconcentrations of CXMplotted against age showa pattern similar to well-established height growth velocity curves and correlate with height growth velocity calculated from incremental height measurements in this study. The CXM marker is stable once collected and can be accurately assayed in serum, plasma, and dried blood spots. CXMtestingmay be useful for monitoring growth in the pediatric population, especially responses of infants and children with genetic and acquired growth disorders to interventions that target the underlying growth disturbances. The utility of CXM may potentially extend to managing other conditions such as fracture healing, scoliosis, arthritis, or cancer.

## INTRODUCTION

Growth is an integral component of human development. Clinically, it typically refers to the skeletal growth measured in infants as body length and as height in children and adolescents. It reflects the dynamic process of endochondral ossification that occurs in growth plates that reside in all bones that contribute to increasing length and height (*1**–**4*).

Growth is often used as a nonspecific indicator of health in childhood (*5*).Most serious illnesses in children are associatedwith reduced growth, which may be restored to normal with successful treatment. Many childhood diseases, typically endocrine disorders, specifically affect growth by affecting hormones and growth factors that regulate bone growth (*6*, *7*). Another large group of childhood growth disorders, the skeletal dysplasias, reflect genetic disturbances in the bone growth machinery (*2*, *8*).

Measuring static parameters of growth, such as body length or height, is relatively simple. In contrast, measuring growth rate or velocity, the key parameter for evaluating and managing growth disturbances, is much more challenging because skeletal growth is a slow process, and measurement techniques lack the precision to accurately detect these small changes. The accepted practice measures length, height, and other anthropometric parameters at 6- or 12-month intervals typically using a calibrated measuring device, such as a stadiometer, and calculates annualized velocity accordingly (centimeters per year) (*9*). Further complicating this approach, especially in infants, are difficulties positioning patients to achieve maximal lengths and completely excluding observer subjectivity.

Despite concerns over the reliability of short-term stadiometerbased height velocity determinations (*10**–**12*), this practice has become established for monitoring the growth of healthy children. Stadiometer-based velocity determination is much less acceptable for managing pediatric growth disturbances, especially for assessing responses to interventions designed to improve growth and health. Thus, there is a clear need for a means to accurately measure growth velocity on a time framemuch shorter than what is currently available.

In this context, we have discovered a marker in the blood of growing infants and children that correlates with growth velocity in real time. This bone growth marker is the noncollagenous 1 (NC1) domain of type X collagen (*13*, *14*). Type X collagen is normally synthesized and deposited in hypertrophic zones of active growth plates and is removed as endochondral ossification proceeds (*15*). Therefore, it is not surprising that the presence of the processed NC1 domain in blood reflects growth plate activity and overall rate of linear bone growth.

We report characterization of the marker, development of enzymelinked immunosorbent assays (ELISAs) to measure it in both mice (designated Cxm for collagen X marker) and humans (designated CXM), as well as the initial analysis of serum, plasma, and dried blood spots (DBS) from normally growing infants and children. Measurement of this marker provides a valuable new tool in the evaluation and management of growth disorders, conditions that disturb normal development, and other clinical situations that involve endochondral ossification.

## RESULTS

### Nature and identification of marker

**Fig. 1:**
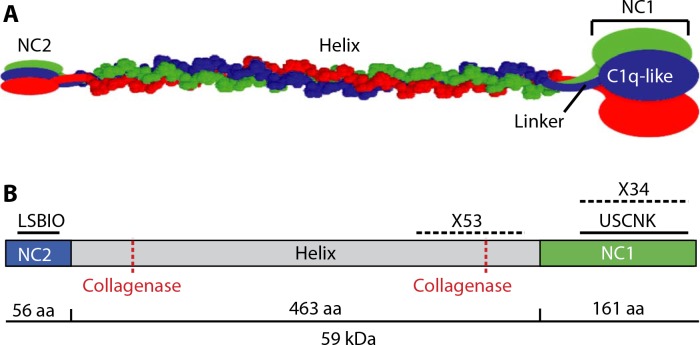
**Depiction ofmammalian type X collagen.** (**A**) Noncollagenous N-terminal (NC2) and C-terminal (NC1) domains are connected by a collagenous triple helix. The NC1 domain is subdivided into a compact “C1q-like” region that resolves in the crystal structure and a linker region that does not. (**B**) Schematic of antibodybinding regions and collagenase sites. Solid lines indicate peptide sequences to which polyclonal antibodies (pAbs) were raised. Hatched lines indicate regions within which X53 and X34 monoclonal antibodies (mAbs) bind. Also shown are two sites susceptible to collagenase cleavage. aa, amino acids.

**Fig. 2:**
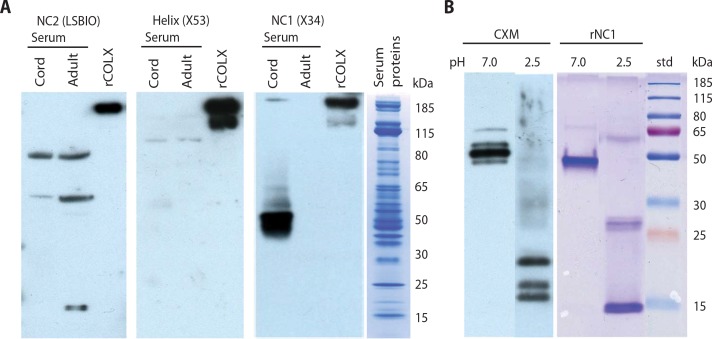
**Identification and subunit characterization of CXM marker**. (**A**) Western blots of umbilical cord serum, adult serum, and recombinant full-length human type X collagen (rCOLX) (positive control). Equivalent blots of 4 to 12% gels were probed with antibodies to the noncollagenous NC2 domain (left panel), collagen helix (center panel), and noncollagenous NC1 domain (right panel). Fourth panel: Representative Coomassie stain of serum proteins present in cord and adult lanes. (**B**) Left panel: Western blot of immunoprecipitated collagen X marker (CXM) eluted at pH 7.0 versus pH 2.5, separated on a 12% gel, and probed with a pAb (USCNK) to the NC1 domain. Right panel: Recombinant trimeric NC1 (rNC1) separated by SDS–polyacrylamide gel before (left lane) or after (right lane) pH 2.5 treatment and stained for protein. std refers to molecular mass standards.

TypeXcollagen is a homotrimeric protein with noncollagenous amine and carboxy termini (NC2 and NC1 regions, respectively) connected by a triple helical collagenous domain (Fig. 1). To identify which of these domains may be present in blood, we compared umbilical cord serum (where type X collagen concentration should be high) to adult serum(where expression should bemuch lower).SDS–polyacrylamide gel electrophoresis (SDS-PAGE)/Western blot analysis of cord versus adult sera was performed after specific immunodepletion of the most abundant serum proteins. Figure 2A shows that recombinant fulllength type X collagen (rCOLX) was detected by the probes for each region, but only the NC1-specific probe monoclonal antibody (mAb) X34 could readily detect proteins in cord serum that were visually absent in adult serum. Because mAb X34 only detects multimeric forms of the NC1 domain (16), the ~50-kDa NC1 region detected in Fig. 2A (third panel)most likely consists of C-terminal trimers. Directly probing blots of serum was considered preferable for this initial screen. However, the high concentration of protein in the serum samples (see the last panel of Fig. 2A) caused the NC1-specific signal to be less well defined compared to affinity-purified samples (Fig. 2B) and produced several nonspecific cross-reactions with the NC2 and helix antibodies.

When the putative marker was immunopurified with immobilized mAb X34, eluted with moderate heat, and probed with a polyclonal antibody (pAb) that recognizes both monomeric and multimeric NC1 regions, the same principal ~50-kDa band was observed (Fig. 2B, left panel, left lane). However, when the immunoprecipitated marker was eluted with acetic acid (~pH2.5) and probedwith the same pAb, lower molecular weight bands of ~17, 19, and 23 kDa were detected (Fig. 2B, left panel, right lane), consistent with their being component subunits of a denaturation-resistant trimeric protein. For comparison, SDSPAGE of recombinant trimericNC1 (rNC1) before and after acetic acid treatment (Fig. 2B, right panel), yielded similar peptides of ~50 and 15 kDa, respectively.

**Fig. 3:**
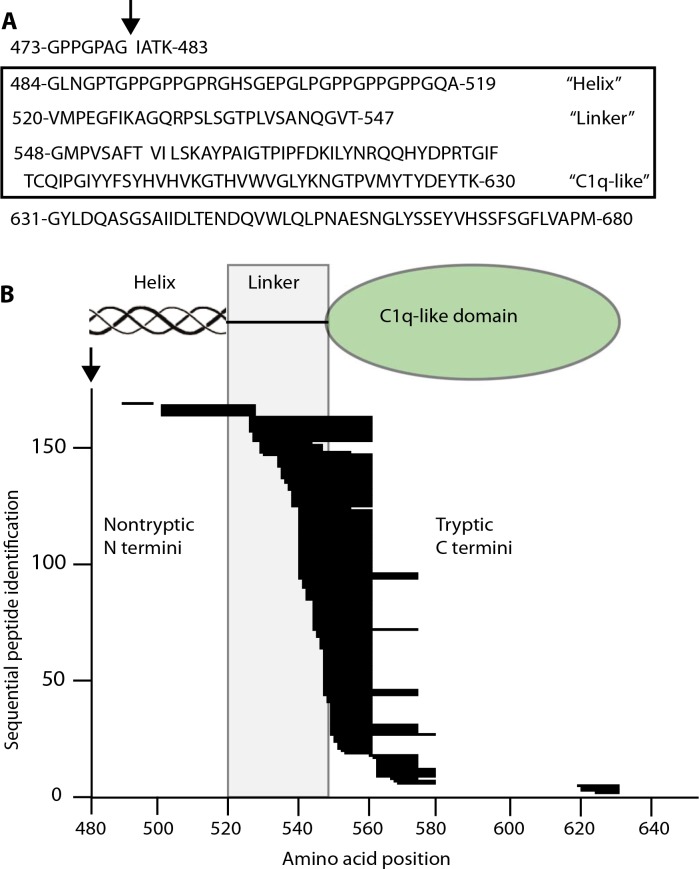
**Mass spectrometry analysis of CXM marker.** (**A**) Boxed area: Region defined by high-confidence peptides identified in mass spectrometry analysis. Above box: Amino acids immediately upstream of identified region that include the proposed collagenase cut site (↓). Below box: The lack of tryptic cut site in C-terminal 50 amino acids makes this peptide too large to identify. (**B**) Semitryptic high-confidence peptide sequences identified by mass spectrometry are represented by stacked horizontal lines corresponding to their placement within the CXM marker. Proposed collagenase cut site (↓) corresponds to amino acid position 480. Functional domains are diagrammed above the graph with the linker region defined by a shaded box. See fig. S1 for the graph of peptides whose N and C termini are both tryptic.

Mass spectrometry of purified/trypsinized marker confirmed its identity. All high confidence sequences mapped from the end of the C1 helix through most of the NC1 domain (G484 to K630, Fig. 3A). The lack of a tryptic cleavage site within the C-terminal last 50 amino acids of type X collagen (G631 to M680) made this peptide too large to be detected. A total of 129 peptides identified resulted from tryptic cleavage at both N and C termini (fig. S1). A total of 168 semitryptic peptides had nontryptic N termini, presumably present in the purified marker before trypsinization (Fig. 3B), whereas only 10 had nontryptic C termini. Most of the nontryptic N termini localized to the 28–amino acid “linker” region between the C1 triple helix and the “C1q-like domain” (*17*). This suggests that the marker is initially released by collagenase activity at a previously proposed site (G479) (*18*) in the C-terminal part of the triple helical domain, just upstreamof the sequence identified here. Additional cleavages then occur in the linker region,whereas the compactly coiled C1q-like trimer resists further proteolysis. The size rangeof suchfragments, containing the entire C1q domain and variable portions of the attached linker and collagenous regions, is consistent with the subunit sizes previously identified byWestern blotting (Fig. 2B; left panel, right lane). Trimers composed of these variably lengthened fragments would then account for themultiple bands shown in Fig. 2B (left panel, left lane).Wedesignated this group of human NC1 trimeric domains with frayed ends as CXM.

### CXM abundance by age and sample source

**Fig. 4:**
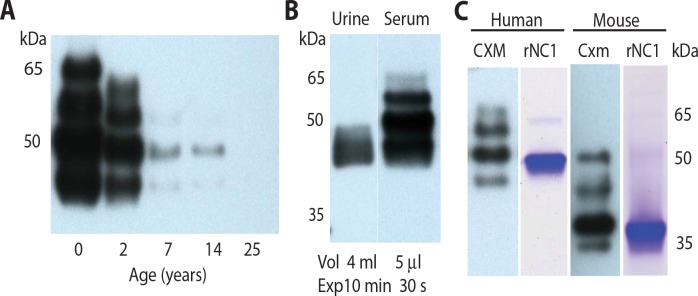
**Marker decreases with age and is detected in human urine and mouse blood**. Western blots of CXM aptoprecipitated with SOMA1and probed with X34 mAb from (**A**) serum of individuals of increasing ages (0 year, umbilical cord serum) or **(B**) matched urine and serum samples from a 2-month-old infant (Vol, volume of sample; Exp, exposure time for autoradiography). (**C**) Aptoprecipitated trimeric markers from human serum (CXM) or mouse serum (Cxm) probed with pAbs raised against their respective recombinant NC1 domains and compared to Coomassiestained gels of the same recombinant proteins (rNC1).

If the occurrence of CXM in blood was an indicator of cartilage turnover in growth plates, then its concentration in blood would be expected to decrease with age as growth velocity slows. Equivalent serum volumes obtained from cord blood (*t* = 0) and subjects who are 2, 7, 14, and 25 years of age were “aptoprecipitated” using a SOMAmer (slow off-rate modified aptamer) (*19*, *20*). Aptoprecipitation is analogous to immunoprecipitation, except that an aptamer reagent (SOMAmer) is used instead of an antibody. This SOMAmer, hereafter referred to as SOMA1, was selected against human rNC1 but recognizes both native human and mouse isoforms. SDS-PAGE/Western blot analysis of the aptoprecipitates was then probed with human-specific mAb X34. Here, the CXM signal dropped progressively with the age of the subject and became undetectable in the 25-year-old adult sample (Fig. 4A); however, the pattern of bands remained the same irrespective of the subject’s age.

An analysis comparing serum and urine obtained from a single 2-month-old infant (Fig. 4B) showed that only low–molecular weight marker components were detected in urine. However, its concentration in urine was ~26,000-fold lower than in serum. In Fig. 4C, the mouse trimeric serummarker (Cxm) showed a pattern of bands similar to the human CXM but migrated about 10 kDa further down the gel. Correspondingly, recombinantmouse NC1, which is trimeric (see fig. S2), showed the same 10-kDa shift. The reason for this mobility difference is not clear; however, the presence of an extra negative charge in the mouse NC1 sequence may contribute.

### Marker analysis in mice ages 1 to 12 weeks

**Fig. 5:**
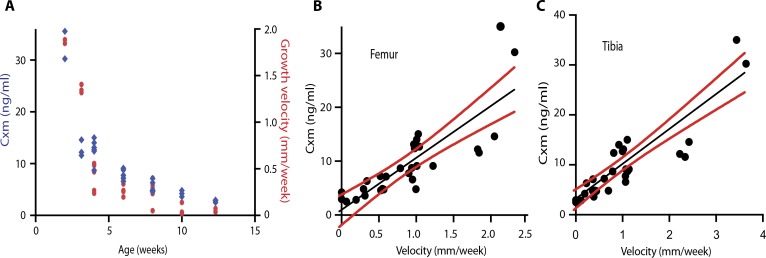
**Correlation of tail and long bone growth velocities with Cxm serum concentrations in mice.** (**A**) Cxm serum concentration (blue) and the growth velocity (red) of mouse tails were plotted against age of mice (n = 29). (**B** and **C**) Cxm serum concentrations were plotted against matched femur (B) or tibia (C) growth velocities (*n* = 29), with linear fit lines in black and 95% confidence intervals in red. Respective Pearson’s correlations are as follows: femur, *r* = 0.82, *P* < 0.0001; tibia, *r* = 0.89, *P* < 0.0001.

The feasibility of using the new marker as an indicator of bone growth velocity was tested in wild-type mice by plotting serum Cxm concentration concentration against age and the growth velocities of the tail, femur, and tibia. Cxm concentration was measured in a sandwich ELISA that used SOMA1 and avian pAb for capture and detection, respectively. Figure 5A shows that Cxm values dropped substantially through the first few weeks in a pattern similar to the decrease in calculated velocity of tail growth. In addition, correlations were obtained when the growth velocities calculated fromfemur and tibia measurements of individual mice were plotted against their Cxm concentrations (Fig. 5, B and C).

### Marker analysis in healthy infants and children

A human CXM ELISA assay similar to the mouse Cxm assay was developed using SOMA1 for capture and mAb X34 for detection. Table S1 summarizes the performance characteristics of this marker assay. Notably, it is sensitive to 5.4 pg/ml (fig. S3), allowing for accurate CXMdeterminations with extremely small volumes of blood, and the CXM marker exhibits stability over a variety of storage conditions (fig. S4). Overall intra-assay coefficient of variation (CV%) of blood samples is on average below 5%, with similarly low interassay variations.

**Fig. 6:**
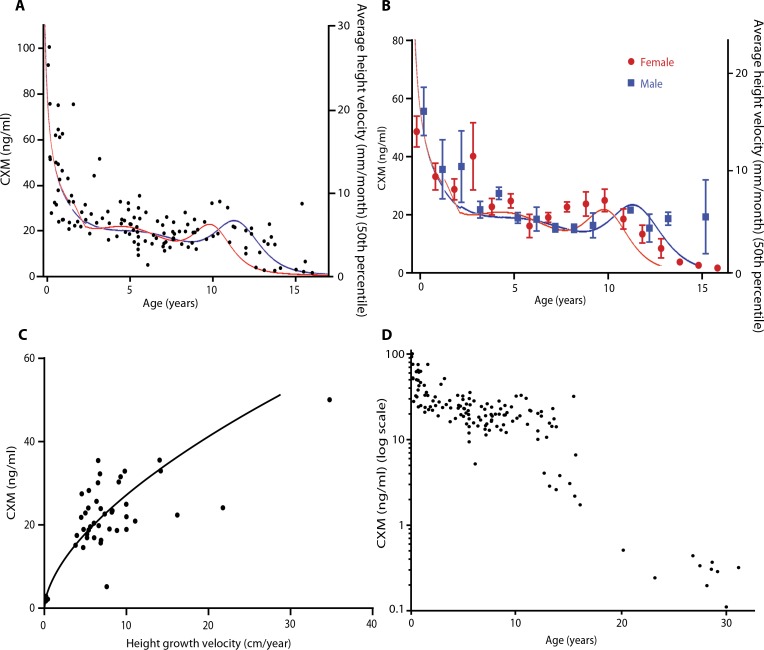
**CXM correlates with age and growth velocity.** (**A**) Serum CXM is plotted against age for normally growing infants and children (*n* = 129). Established height velocity curve averages for males (blue line) and females (red line) are superimposed for comparison. (**B**) CXM is plotted against age, grouped by sex, and shown as means ± SE. Sex-matched velocity norms for males and females are superimposed as before. (**C**) Infants and children 0.18 to 16 years of age were measured for length/ height and assayed for serum CXM at 0-, 6-, and 12-month periods (*n* = 44). Height velocities were calculated as change in length/height over time interval, converted to centimeter per year, and plotted against CXM [adjusted *R* ^2^ (weighted) = 0.88, *P* < 0.001). (**D**) Log-transformed CXM serum concentrations for normally growing children and nongrowing adults are plotted against age (*n* = 139).

In accordance with local Institutional Review Board approval and after the nature and possible consequences of the studies were explained, serum samples obtained from 83 normally growing, healthy infants and children ranging in age from birth to 18 years were assayed for CXM and compared (Fig. 6A). To maximize sample size, we relaxed the assumption of independence and included observations for normally developing children who were measured two or three times (mean, 2.125) at 6-month intervals (*n* = 40) along with 43 normally developing children and 10 adults who were measured once. Established growth velocity curves for infants and children of both sexes are superimposed on Fig. 6 (A and B) for reference (*21*). Male and female CXM concentrations were not statistically different when prepubertal age groups were compared (Fig. 6B). However, the concentrations varied more during pubertal years and differed between males and females, presumably reflecting the variability in timing of pubertal growth spurts. These cross-sectional data document that CXM concentrations parallel well-established growth velocity standards that are commonly used to evaluate childhood growth.

### Human growth velocity measurements

Longitudinal height data and blood samples collected at about 6-month intervals from 26 individuals allowed CXMconcentration to be plotted against annualized height velocity (Fig. 6C). To maximize sample size, we relaxed the assumption of independence and included two growth velocity observations for 14 children along with 12 with only one observation. A nonlinear power series algorithmwas used to fit data with the respective coefficient of determination shown. The linear correlation of CXM and height velocity was more modest in this sample (Pearson’s *r* = 0.66; P < 0.001; 95% confidence interval, 0.45 to 0.80) than in the mouse samples, but fitting a nonlinear power series line improved the correlation of our marker to height velocity in humans [adjusted *R*^2^ (weighted) = 0.88]. The observed association is consistent with our model that the concentration of the marker reflects growth plate activity and the rate of skeletal growth; however, the sample size was too small to confidently fit a curved function to the data.

To document that our study population was growing normally, we plotted stadiometer-based height velocities of 23 subjects between the ages of 3.3 and 9.5 years against established norms for this age group (fig. S5A) (*22*). This age range was used because growth is typically relatively steady. With exception of two subjects who plotted slightly beyond 2 SDs, our subjects fell within 2 SDs of the norms, indicating that our population was not skewed.

It is difficult to directly compare CXM-based estimates of height velocity to stadiometer-based (observed) height velocity determinations because they measure different parameters of growth. To gain insight into this issue, we plotted CXM values and observed height velocities against age and visually compared their relative dispersion (fig. S5, B and C). This comparison showed less dispersion for the observed velocity measurements than CXM, suggesting that observed measurements may be better for accurately determining height velocity averaged over several months; however, it is unlikely that CXM would be used for this purpose.

### CXM in healthy adults

In contrast to growing children, CXM concentrations dropped to around 300 pg/ml on average in adults. To show the full range of CXM values, we plotted CXM concentrations from 10 healthy, nongrowing 20- to 30-year-old adults on a logarithmic scale with the younger subjects previously mentioned (Fig. 6D). CXM appears to level off in healthy adults at concentrations well below those of growing children.

### CXM in adult fracture healing

**Fig. 7:**
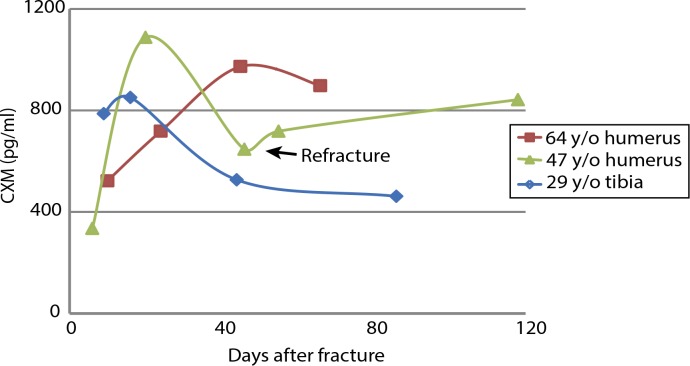
**CXM concentration increases during adult fracture healing.** Plot showing CXM concentration measured at different time points after acute long bone fractures in a 29-year-old male and in 47- and 64-year-old females. Arrow indicates refracture in the 47-year-old patient.

Bone fractures heal through endochondral ossification during which type X collagen–containing fracture callus is degraded and replaced by bone, similar to what occurs in the growth plate (*23*). The rate of healing and amount of callus vary by fracture severity, how well the healing fracture is stabilized, and the size of bone that is fractured. Most likely, the relative amount of CXM released from a single or even a few fractures would be less than the amount released from all growth plates in a growing skeleton, so our assay would be unlikely to detect minute changes in CXM concentrations in children with fractures. In adults, low endogenous concentrations of CXM may allow for monitoring fracture healing using the CXM marker. Preliminary evidence shows that a temporal pattern in which CXM rises, peaks, and then falls during fracture healing can be detected in adults (Fig. 7). This temporal pattern is consistent with the “endochondral” phase of fracture healing, which typically occurs from 1 to 3 weeks after initial fracture. The 47-year-old female subject in this figure offers a unique window into the proposed relationship between CXM and fracture healing. This individual’s initial fracture was associated with a peak in CXM at 20 days after fracture, but she then experienced a proximal refracture, which was associated with another rise in CXMthat corresponded temporally to the radiographic evidence of secondary fracture callus. The comparison of the temporal patterns of CXMduring fracture healing of the 64-year-old versus the 29-year-old subjects is consistent with the notion that healing may occur more slowly with aging (*24*, *25*).

### Serum versus plasma versus DBS

Our marker ELISA was developed using serum; however, inmany instances, only plasma or DBS samples are available, which have been shown to give equivalent results in other marker assays (*26*). To determine the suitability of these alternative blood samples for CXM, we compared concentrations of the marker in subjects whose blood was collected as serum and plasma or serum, plasma, and DBS simultaneously. Eighty paired serum and plasma samples were collected and assayed, and CXM results for plasma showed slightly higher values on average (+7%) compared to their paired serum counterparts (table S1 and fig. S6).

When comparing paired serum versus DBS or plasma versus DBS samples, the matched concentrations suggest that DBS may be more comparable to plasma rather than serum. The Pearson’s r for plasma versus DBS was better than that for serum versus DBS at 0.92 versus 0.84, respectively. DBS average readings tended to be higher on average with higher variability versus both serum and plasma. Given the potential variations inherent in DBS sampling procedure and extraction compared to venipuncture, it is not surprising thatwe observed more variability with our DBS samples. Despite these variability issues, analysis of the extracted DBS gave comparable results to our matched serum and plasma samples (fig. S6).

### Biologic variation

**Fig. 8:**
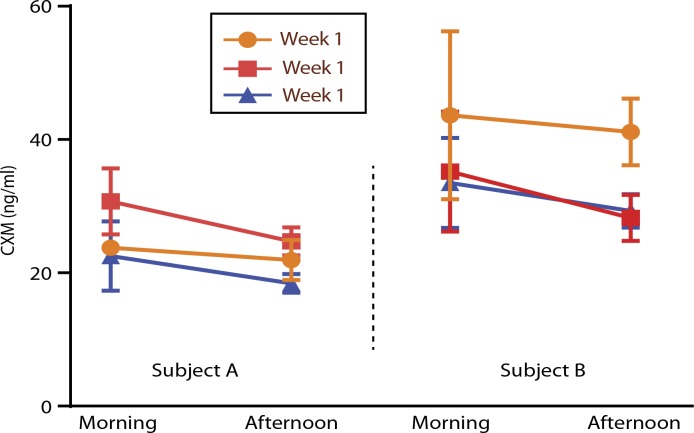
**Diurnal variation of CXM.** Morning and afternoon CXM concentrations from dried blood spots for different aged children. Subject A: a 4-year-old female tested morning and afternoon for three consecutive weeks (*n* = 27). Average CXM readings were plotted. Subject B: an 11-year-old female tested morning and afternoon for three consecutive weeks (*n* = 28). Average CXM readings and SD are plotted.

Many markers exhibit diurnal variation. To determine whether CXM shows such variation, we measured CXM in 12 normally growing children ages 2 to 14 years with well-controlled diabetes. DBS cards were spotted, and the time recorded was coincident with finger stick for glucose monitoring. Sampling was at least three times a day for three consecutive days and in some cases for three consecutive weeks. Using 2 p.m. as cutoff for morning and afternoon samples, CXM concentrations were on average 26% higher before 2 p.m. than after 2 p.m. (data shown in table S2). Figure 8 illustrates this pattern, and modest weekly variation is shown fromtwo girls sampled over 3 weeks.

To assess the stability ofCXM/Cxmin the circulation,mouse rNC1 was injected intravenously into 25-week-old mice, and blood samples were assayed at various times up to 240 min after injection (fig. S7). The results suggest that CXM/Cxm has a half-life of about 30 min.

## DISCUSSION

Our results suggest strongly that CXM, the intact trimeric NC1 domain of type X collagen, escapes degradation in the skeletal growth plate and can be detected in blood, where its concentration reflects overall growth plate activity in the body and correlates with velocity of skeletal growth. Hence, this degradation by-product of skeletal growth behaves as a real-time marker for linear skeletal growth velocity and has many potential clinical applications.

### CXM identification, characterization, and assay

The synthesis of type X collagen is normally restricted to the hypertrophic zone of the skeletal growth plate, where it is secreted into cartilage matrix during the latter stages of endochondral ossification in all growing bones. This matrix serves as a template for bone growth during which it is degraded as growth proceeds until growth stops after adolescence. The interface between the hypertrophic zone and newly formed bone—ossification front—is highly enriched in extracellular proteolytic enzymes engaged in degrading and removing hypertrophic cartilage matrix as the ossification front expands and the bone lengthens. The enzymes known to have collagenase activity, which are thereby candidates for type X collagen degradation, include matrix metalloproteinase 13 (MMP13) secreted from terminally differentiated hypertrophic chondrocytes, MMP9 secreted from osteochondroclasts, and proteases released from vascular cell precursors that invade the cartilage template from the bone marrow (*27*).

Type X collagen has two proposed collagenase cleavage sites in its helical domain (Fig. 1). The ~50-kDa size of the predominant fragment detected by Western blot suggests that CXM is the product of the carboxy collagenase cleavage plus additional cleavage events that trim the fragment to smaller sizes. Themouse Cxmappears to undergo cleavages similar to the human CXM. Detection of distinct bands slightly larger and smaller than the predominant 50-kDa human CXMband combined with the mass spectrometry results implies that there are favored cleavage sites at the N-terminal end of the C-terminal collagenase cleavage fragment. Our attempts to identify the cleavage sites by N-terminal sequencing have been unsuccessful to date.

Our mouse studies suggest that the CXMmarker in vivo half-life is relatively short (~30min). In contrast, themarker is very stable in vitro, in isolated serum, plasma, and DBS samples. For example, CXM displays <10% degradation in serum for 18 hours at 37°C (fig. S4), can undergomultiple freeze thaws, and resists degradation at temperatures above freezing point. The ability of the marker to resist proteolysis likely reflects its compact molecular configuration (*17*). CXM’s resistance to serum proteases and low urinary excretion suggests that another clearance pathway is involved. Trimeric adiponectin, a circulating hormone that is both genetically closely related to type X collagen and structurally similar toCXM, is rapidly cleared by the liverwith a very similar half-life (*28*), suggesting that CXM may be removed through a similarmechanism, although themechanismwas not investigated here and will require further study.

Analysis of paired serum, plasma, and DBS samples showed that CXM concentrations were similar across sample types, although plasma and DBS readings tended to be slightly higher on average than serum values (table S1). Differences in marker concentrations have been shown in matched biological sample types, so this result is not surprising (*29*). The DBS determinations were on average closer to those of the plasma samples rather than serum, suggesting that plasma may be the preferred choice of blood specimens for this assay in the future, but more definitive studies will be needed to resolve this question. Notably, the overall inter- and intra-assay variations of plasma and serum samples were comparable, but DBS samples varied more, perhaps reflecting variability in sampling technique and DBS handling.

### Clinical relevance

It is well established that growth velocity is highest in young infants, drops substantially over the first 2 to 3 years, remains relatively low during childhood, increases modestly during the pubertal growth spurt, and drops to zero after the spurt is complete. The scatterplot of our cross-sectional serum data from healthy infants and children shows a similar trend (Fig. 6A). Our numbers are preliminary in that they represent the first attempt to relate CXM to established human growth data, but they provide strong support for our hypothesis that the marker levels reflect skeletal growth velocity.

We must emphasize that CXM presumably represents a real-time readout of growth plate activity that corresponds to instantaneous skeletal growth velocity at the time of sampling in contrast to average growth velocity calculated from measuring incremental growth over several months, typically 6 months or more. Hence, no comparable marker exists for CXM validation. If growth were a slow, steady, and constant process, then onewould expect the real-time and average velocities to be very similar. However, if growth varies from day to day or even by time of day, as our preliminary data suggest, then the two might not agree. Similarly, CXMdoes not necessarily predict length or height, both of which reflect accumulated growth in contrast to CXM, which measures growth rate at a single point of time. Despite these caveats, both mouse Cxm and human CXM values correlate with velocities calculated from measured interim growth, suggesting that variability must not be too great.

The correlation ofCXMto growth velocity in human subjects was higher using a nonlinear power curve [adjusted *R*^2^ (weighted) = 0.88, *P* < 0.001] rather than a linear best fit (Pearson’s *r* = 0.66, *P* < 0.001) that was used with the mouse data. Figure 6 included some participants with more than one data observation. The relaxation of the assumption of independence might lead to narrower sample variability and risk modest inflation of the association of CXM and growth velocity. With a larger data set, it may be found that a linear fit is more appropriate for plotting growth velocity versus CXM concentration; however, the strong correlation from our data set demonstrates that CXM has the potential to provide estimates of growth velocity with narrow margins of error. Further investigation can clarify whether the relationship of CXM to growth velocity is linear or curvilinear and whether that varies within certain ranges of growth velocity. Accordingly, CXM appears to be an informative, real-time indicator of skeletal growth velocity that has considerable potential benefit for the clinical management of skeletal growth and its disorders.

We anticipate that CXM-based estimates of height velocity will be compared to conventional stadiometer-based height velocity determinations. Accordingly, we must emphasize that they measure different parameters of growth, instantaneous growth velocity versus growth velocity averaged over 6 to 12months, respectively. Consequently, they have different clinical applications and different utilities. For example, stadiometer-based methods will be most useful for cross-sectional, long-term studies. In contrast, we predict that CXM measurements will be most useful for assessing responses of individual children to interventions that affect growth in days to a few weeks. The difference is analogous clinically to the difference betweenmeasuring serumglucose and hemoglobin A1c in diabetic patients. The former measures glucose concentration at the time of sampling; the latter is an indicator of glucosemetabolism over ~3months (*30*). Both are used in themanagement of diabetes but for different purposes; the utility of one marker does not diminish the utility of the other.

The most obvious practical application of the CXM marker is for monitoring the growth response of poorly growing infants and children to interventions designed to improve growth. Examples include growth hormone and C-type natriuretic peptide derivative therapies for infants and children with growth hormone deficiency and achondroplasia, respectively. Compared to cross-sectional studies, the infant or child serves as his/her own control in this setting, minimizing person-to-person variation. It is likely that treatments that directly or indirectly improve growth begin to act on the bone growth machinery within days or a few weeks at the least and that resulting changes in growth velocity could be detected by measuring CXM within this time frame assuming that baseline concentrations were determined. Information about how an infant/child responds to treatment amonth after initiation would be a substantial advantage over the current practice of waiting 6 months or more for growth velocity information. Being able to detect responses to therapeutic interventions in a much shorter time frame would greatly facilitate the adjustment and comparison of therapeutic interventions in these instances. It would also provide a new tool to investigate in depth how the skeleton responds to growth-promoting interventions. Similarly, CXMtesting may facilitate the assessment and comparison of the efficacy of programmatic interventions developed to alleviate malnutrition and other chronic diseases that negatively affect growth in resource-restricted regions of the world.

Our testing of healthy diabetic children suggests thatCXMexhibits diurnal variation with values highest in the morning, which would be consistent with the notion that diurnal factors, such as growth hormone, drive bone growth (*31*). Alternatively, diurnal variation of CXM could simply reflect loading (rising from bedtime horizontal position to daytime upright stature forces CXMfrom the growth plate into subchondral blood vessels). Further investigation is needed to resolve the nature of apparent diurnal variation; however, in the interim, it seems prudent to sample blood in the morning if CXMis being used to monitor growth of a child over time. Similarly, additional investigation will likely refine the optimal conditions for measuring and interpreting CXM values as is typical after the initial identification of a new diagnostic marker.

Lampl and colleagues (*32*) have proposed that growth occurs in an episodic or saltatory fashion in short-duration, high-velocity spurts separated by periods of little or no growth. Others have argued against this model (*33*). Our data showing ~20% average variation in day-today and week-to-week CXM levels seem less than one would expect for the marked changes in growth predicted by the saltatory growth model. If confirmed by further investigation, then our preliminary observations would be most compatible with small to modest variation in growth velocity from day to day. CXMcould serve as a valuable tool to investigate short-termvariations in bone growth and their relationship to conventional parameters of growth.

Many of the growth plates that contribute to blood CXM values may not contribute to skeletal length or height, so one might argue that linking it to linear growth may not represent a perfect correlation. However, we believe that the largest and most active growth plates in the body, namely, those in the proximal and distal femurs and tibias, as well as the less active growth plates of the vertebral bodies, are likely to contribute most of the measurable CXM. Moreover, the correlations that we detect for CXM versus length/height velocity and remarkable similarities of plotting CXM versus age to curves that plot clinically determined growth velocity to age argue that CXMis a useful indicator of linear bone growth.

The CXMmarker has potential applications beyond those directly related to bone growth. For example, the management of idiopathic scoliosis frequently involves bracing and surgical fusion of the spine (*34*). In both cases, the timing of intervention depends on the timing of the pubertal growth spurt; bracing takes advantage of the spurt, whereas surgical fusion is done after the spurt is finished. Frequent CXM testing could be used to guide the timing of both interventions.

Long bone fractures heal through endochondral ossification during which type X collagen–containing fracture callus is degraded and replaced by bone much like that which occurs in the growth plate, although the rate is influenced by other factors such as fracture severity, site, and stabilization (*23*). The data shown in Fig. 7 are clearly preliminary, but they show that CXM concentrations increase temporarily during the time frame when fractures would be expected to heal. They also lend evidence to the fact that our assay is sensitive enough to detect small changes over baseline CXM levels in adult subjects. Furthermore, these data support the concept that CXM is an indicator of endochondral ossification.

Articular chondrocytes often terminally differentiate (hypertrophy) in osteoarthritis (OA), raising the possibility that type X collagen could be used as a marker of OA activity (*35*). Low levels of type X collagen have been detected in sera from adults with severe OA (*36*). The reported concentrations (24 to 128 pg/ml) are about three orders ofmagnitude lower than those that we detect in growing infants but within the detectable limits of our assay. The epitope for the assay developed by these investigatorsmaps to the NC1 domain of type X collagen. It is possible that the mAb reported in this publication detects the same NC1 fragment reported here, although no biochemical studies were done to characterize the antibody target.

Type X collagen has been linked to cancer in two publications. In one case, it was detected by ELISA in sera of adult patients with colon cancer (*37*). The authors speculated that Runx2, a known transcriptional regulator of COL10A1 expression, is responsible for type X collagen production in the tumors. The second report detected expression of COL10A1 mRNA by microarray analysis in diverse cancer types but not in normal tissues (*38*). Immunostaining of breast cancer tissues localized it to blood vessels, suggesting that its expression is associated with vascular invasion of tumors. These reports raise the possibility that CXM could also be used as a marker for cancer detection in adults, but this will require further investigation.

## MATERIALS AND METHODS

### Study design

The goals of this project were to determine whether type X collagen or a fragment of type X collagen could be used as a marker for bone growth velocity and, if so, develop assays to measure it in children. The first goal was addressed using a biochemical approach; the second goal was addressed by development of ELISAs to detect and quantify the marker in blood samples from humans and mice.

All serum, plasma, and DBS samples were collected prospectively under protocols approved by the Institutional Review Board from children and adults between 2014 and 2016 from either Shriners Hospitals for Children or fromOregonHealth and ScienceUniversity (OHSU) in Portland, OR. Patients from Shriners Hospitals for Children were enrolled for single appointments, where serum, plasma, DBS, and urine samples were collected at the same time. Patients from OHSU were enrolled in a longitudinal study collecting serum and DBS at time points of about 0, 6, and 12months. Sample sizes for tests ofmarker to growth velocity associations were determined by a priori power analyses using standard values for type I error (α = 0.05) and type II error (β = 0.2; hence, power 1 − β = 0.8) to detect correlations of 0.4 or larger.

Heights were measured on an easy glide stadiometer (Perspective Enterprises) calibrated by a standard 100-cm rod. Measurements were done in a clinical setting in the Pediatric Endocrine and Diabetes clinics by a medical assistant specifically trained in accurate measurement techniques. Umbilical cord blood samples were obtained through the Oregon Cord Blood Donation Program at OHSU. Umbilical cord serum samples were purchased from BioReclamationIVT. Height, weight, and arm span measurements were recorded at the time of sampling for each patient. Growth velocity was calculated using the change in height measurements from longitudinal samples collected. Plasma and serum samples were processed in Vacutainers (Becton Dickinson #368036 and #367983, respectively), aliquoted into microcentrifuge tubes, and stored immediately at −20°C. DBS samples were obtained by finger sticks and spotting onto Whatman 903 Protein Saver cards. DBS cards were then dried for 1 to 4 hours at room temperature, placed in resealable bags containing desiccant packets, and stored at −20°C until assayed.All samples included in this study were assayed in a blinded fashion in duplicate. Information pertaining to these samples can be found in table S3.

Samples for diurnal variation testing were obtained from well-managed but otherwise healthy diabetic children ages 2 to 14 years who were enrolled in the OHSU Pediatric Diabetes Clinic. Patients prepared a DBS each time they stuck their finger for glucosemeasurements. The time and date were recorded, and dried cards were stored desiccated in a resealable bag in the dark at room temperature. Once sample collection was completed, the cards were returned to Shriners Hospitals for Children in envelopes satisfying mailing requirements provided by the Center for Disease Control and Prevention. Upon arrival, DBS cards were stored at −20°C until assayed.

Samples for fracture testing were collected at the University of California, San Francisco (UCSF) Zuckerberg San Francisco General Hospital and Trauma Center. Fracture patients were enrolled within 2 weeks of experiencing a fracture. The fractures were documented radiographically, and DBS samples were collected at initial appointment and at each checkup thereafter. DBS cards were then dried for 1 to 4 hours at room temperature, placed in resealable bags containing desiccant packets, and stored at −20°C. DBS cards were mailed in dry ice packages to Shriners Hospitals for Children in Portland, OR for CXM concentration testing using standard DBS elution and testing protocols.

### Recombinant proteins

Recombinant proteins to human and mouse NC1 regions were from BioMatik (human rNC1 #RPU140912, mouse rNC1 #RPU140913). Recombinant peptides had a polyhistidine tag (MGHHHHHHSGSEF) followed by the NC1 protein sequences: human, TGMPVSAFTVILSKAYPAIGTPIPFDKILYNRQQHYDPRTGIFTCQIPGIYYFSYHVHVKGTHVWVGLYKNGTPVMYTYDEYTKGYLDQASGSAIIDLTENDQVWLQLPNAESNGLYSSEYVHS SFSGFLVAPM; mouse, TGMPVSAFTVILSKAYPAVGAPIPFDEILYNRQQHYDPRSGIFTCKIPGIYYFSYHVHVKGTHVWVGLYKNGTPTMYTYDEYSKGYLDQASGSAIMELTENDQVWLQLPNAESNGLYSSEYVHS SFSGFLVAPM.

### Type X collagen antibodies

Human-specific mousemAbsX34 andX53 (*16*)were either conjugated to horseradish peroxidase (HRP, SouthernBiotech) or covalently coupled to agarose using AminoLink Plus Immobilization Kit (Thermo Fisher Scientific, #44894). Rabbit pAbs were raised against both human andmouse rNC1 (USCNK, #PAC156Hu01 or #PAC156Mo01) or a humanNC2 peptide (LifeSpan BioSciences, #LS-C157654). Aves Labs Inc. prepared and purified a chicken pAb to the mouse rNC1 sequence above. HRP-conjugated secondary antibodies included goat anti-rabbit (Amersham,#NA934V) and goat anti-chicken (Aves Labs Inc., #H-1004).

### Components for ELISAs

The following were used: 96-well high-binding plate (Costar, #3590), immunopure streptavidin (Thermo Fisher Scientific, #21125), Super- Block Blocking buffer (Thermo Fisher Scientific, #37515), bovine serum albumin (BSA) for coating plates (RMBIO, #BSA-BAF-01K), BSA for assay solutions (Gold Biotechnology, #A-421-100), Tween 20 (Thermo Fisher Scientific, #BP337-500), and dextran sulfate sodium salt (Sigma-Aldrich, #31404-25G-F). Calibrators for assays were rNC1 proteins from BioMatik as described above.

### ELISA buffers

The following buffers, reagents and diluents for ELISA are as follows: SOMA1 buffer with Tween 20 (SBT) [100 mM NaCl, 5 mM KCl, 10 mM hemisodium Hepes (pH 7.5), and 0.05% Tween 20]; SBT bufferwith 5mMMgCl_2_ (SBTM); SBTbufferwith 5mMEDTA(SBTE) sample diluent (SBTM buffer + 1% BSA and 1% dextran sulfate); conjugate diluent (SBTM buffer + 1% BSA); coating buffer [1.59 g of Na_2_CO_3_/2.93 g of NaHCO_3_ in 1 liter of H_2_O (pH 9.6)], phosphate buffered saline with Tween 20 (PBST); blocking buffer (PBST + 1% BSA); SOMAmer plating buffer (SBTE + 1% BSA); and stop solution (160 mM H_2_SO_4_).

### Other buffers

Other buffers that were used are as follows: Tris buffered saline with Tween 20 (TBST); gel-loading buffer [sample buffer (Thermo Fisher Scientific, #NP0007) + sample reducing agent (Thermo Fisher Scientific, #NP0009)]; low-salt buffer [1 mMHepes (pH 7.5), 1 mMMgCl_2_, and 02% Tween]; and SOMAmer elution buffer [20mMethanolamine (pH 10), 5 mMEDTA, and 0.02% Tween].

### Other components

The following were used:AmiconUltra centrifugal filters (#UFC200324), Pierce streptavidin magnetic beads (Thermo Fisher Scientific, #88816), Bolt Antioxidant (Thermo Fisher Scientific, #BT0005), Imperial protein stain (Thermo Fisher Scientific, #24615), Pierce Top 12 Abundant Protein Depletion Spin Columns (Thermo Fisher Scientific, #85165), AminoLink Plus Immobilization Kit (ThermoFisher Scientific, #44890), NuPAGE Bis-Tris and Tris-Glycine gels (Thermo Fisher Scientific), human adiponectin (R&D Systems, #1065-AP-050), human C1q (Abcam, #ab96363), human collagens type I and II (Abnova, #P4915 and #P4916), and human collagen typeVIII a1 anda2NC1domains (Antibodies Online, #ABIN1079239 and #ABIN1098982).

### Identification of marker in “depleted” cord serum

After depletion of their most abundant serum proteins (using Thermo Fisher Scientific, #85164 columns), umbilical cord and adult serum samples were concentrated on 3-kDa ultra-centrifugal filters and loaded on a 4 to 12% bis-tris gradient SDS-PAGE gel system (5 μl of serum per lane). Full-length type X collagen from the medium of a human embryonic kidney cell line developed by Wagner et al. (*39*) was used as a positive control. The separated proteins were transferred to nitrocellulose at 56 V for 1 hour, blocked in TBST + 3% BSA for 1 hour, and probed withHRP-X34 (anti-NC1) andHRP-X53 (anti-C1) at a 1:5000 dilution or a polyclonal anti-NC2 at 1:1000 dilution followed by an HRP-conjugated secondary antibody. Antibody incubations were in TBST + 1% BSA for 1 hour.

### Immunoprecipitation, aptoprecipitation, and Western blot procedures

All precipitations were performed overnight at 4°C with end-to-end turning. Immunoprecipitation with mAb X34 agarose (10 μl of 50% slurry for each 5 μl of serum) was performed in PBST, after which the agarose beads were washed 5× in the PBST. Trimeric marker was eluted from mAb X34 by moderate heating in gel-loading buffer (70°C for 10 min). Monomeric subunits were generated by eluting beads with 100 mM acetic acid (~pH 2.5) followed by lyophilization of the eluate and resuspension of protein in gel-loading buffer. Aptoprecipitations were performed with SOMA1-magnetic beads (2.6 nmol of biotinylated SOMA1/10 mg of streptavidin magnetic beads) using 5 μl of a bead solution (10 mg/ml) per 5 μl of serum diluted into SBTM. Beads with bound markers were washed 3× with SBTM and eluted in a small volume of SOMAmer elution buffer (pH 10) before adding to gel-loading buffer. After SDS-PAGE, proteins were transferred to nitrocellulose at 56 V for 1 hour at 4°C. The blots were then blocked with 3% BSA in TBST, washed, and probed as described.

### Purification of marker

Cord plasma was obtained after centrifugation of donated cord blood samples, and each unit received 4.18 ml of 1 M MgCl_2_, 2 ml of 1 M hemisodiumHepes, and 2ml of 100mMsodiumEGTA. Then, 6 ml of 10% dextran sulfate was added slowly with stirring to prevent formation of aMg^++^/dextran sulfate precipitate. This preparation was placed on ice, stirred slowly for 1 hour, and spun at 8000g for 1 hour. The resulting supernatant was distributed into 50-ml tubes, with 1.7 mg of SOMA1-magnetic beads (see above) per tube. The tubes were turned end over end overnight, after which the magnetic beads were collected into 1.5-ml conical tubes and washed sequentially with SBTM (3 × 1 ml), SBTM+ 4MNaCl (4 × 1ml), SBTM(1 × 1ml), and low-salt buffer (2 × 1 ml). Elution of CXM was performed by adding 100 μl of SOMAmer elution buffer to the pooled beads and shaking on an orbital mixer for 10 min at room temperature. The resulting supernatant was highly enriched in CXM in its native trimeric form. At this point, four volumes of SBTM with elevated Hepes (50mM/pH 7.5) were added to neutralize the sample for long-term storage.

### Mass spectrometry

CXMwas purified from six units of cord plasma (~250ml) according to the procedure described above. To concentrate, denature, and dissociate CXM subunits, 400 ml of the marker in SOMAmer elution buffer was directly precipitated with 10% trichloroacetic acid, acetonewashed, and dried for 10 min at 96°C. The dried pellet was dissolved in 20 µl of gel-loading buffer and heated at 96°C for 10 min. Two lanes of a 12% NuPAGE Bis-Tris gel were loaded for SDS-PAGE (Bolt Antioxidant was added to the upper-tank buffer to reduce in-gel oxidation). One lane, containing 5% of the sample, was subsequently blotted and probed with the anti-NC1 USCNK pAb to determine the position of CXMon the gel. The other lane, containing the remaining 95%, was directly stained with colloidal Coomassie blue, and the corresponding region was excised. This gel fragment was digested with ProteaseMax + trypsin and analyzed on a Thermo Fisher Scientific Orbitrap Fusion Mass Spectrometer. Collagen X peptides were identified using the SEQUEST data analysis program (*40*). Data analysis was performed within the Proteome Discoverer software suite (Thermo Fisher Scientific). SEQUEST HT was used to search tandem mass spectrometry spectra against a June 2016 version of the human SWISS-PROT database, and the percolator-filtered resulting peptide matches to an overall false discovery rate of 1%. The 307 high-confidence identifications of type X collagen presented had an average cross-correlation (XCorr) of 3.5 and an average Dmass of 0.79.

### Development of SOMAmer capture reagent for CXM

The recombinant human NC1 region described above was biotinylated and submitted to SomaLogic Inc. for “SELEX” affinity capture (*41*) of potential high-affinity SOMAmers. Figure 2B indicates that the recombinant peptide was in its native trimeric form. Before performing SELEX selection, the following proteins were preadsorbed to the SOMAmer library to avoid potential cross-reactivity: human collagen types I, II, and VIII and the serum proteins adiponectin and complement C1q. Ten high-affinity SOMAmers were generated, of which the highest affinity form (SOMA1; 160 pM) gave the best response when used in a sandwich assay with HRP-conjugated mAb X34.

### Assay procedure

1) Sample incubations. Calibrators, controls, and samples were prepared in sample diluent and aliquoted into “SOMA 1 capture” assay plates (see the Supplementary Materials). All sample, detector, and reporter incubations were at 100 ml per well and performed at 37°C with shaking at 450 revolutions per minute (rpm).

2A) Human assay detector incubation. Plates were washed 3× with SBTM, patted dry, and incubated with HRP-conjugated mAb X34 (1:5000 in conjugate diluent) for 1 hour. 2B) Mouse assay detector/reporter incubations. Plates were washed 3×with SBTMand incubatedwith chicken anti–mouse-rNC1 (5 mg/ml in conjugate diluent) for 1 hour. Plateswerewashed 5×with SBTMand incubated with HRP-conjugated secondary antibody (1:5000 dilution in conjugate diluent) for 1 hour.

3) Develop and read. Plates were washed 3× with SBTM, tapped dry, and developed with a tetramethylbenzidine (TMB) substrate at room temperature. After 10 min, the reaction was stopped by adding 50 μl of stop solution and brief mixing on a shaker at 650 rpm.The optical density at 450nmwas readwithin 30min of stop solution addition.

### ELISA assay calibrators and controls

The rNC1 from BioMatik was reconstituted as per instructions. Absolute concentration was initially determined using a Qubit 2.0 Fluorometer from Invitrogen and confirmed by amino acid analysis using a Hitachi L-8800A. Calibrators were prepared by diluting rNC1 to sample diluent (800 pg/ml) and serial dilution (12.5 pg/ml). Quality controls (QC) were created by diluting rNC1 into sample diluent to concentrations of 700, 250, and 10 pg/ml, respectively. Serum and plasma samples from normally growing children were diluted 1:200 in sample diluent. Quality control of interassay and intra-assay determinations was monitored using matrix-specific (serum, plasma, or DBS) rNC1-spiked controls at low, medium, and high concentration levels along with full calibration curves for each ELISA plate. Assays were deemed valid if QC replicates were <20% intra-assay CV% and within ±20% of interassay assigned concentration [except for rNC1 QC (10 pg/ml) (low) due to its low concentration].

### DBS elution procedure

One 3.1-mmpunch was taken per pediatric DBS spot and eluted with 250 ml of sample diluent in the well of a sealed polypropylene microplate. Because of low CXM concentration, adult samples used two punches. The plate was incubated overnight at 4°C on ice to reduce condensation. Finally, the elution plate was then placed on a shaker at 450 rpm for 10 min at room temperature. Each sample (100 ml) was assayed in duplicate, and the concentration was determined from a serially diluted rNC1 calibrator curve using four-parameter logistic nonlinear regression model fit from BioTek Gen5 software (*R* ^2^ > 0.95 was acceptable). DBS quality controls of 70, 30, and 1 ng/ml were also added to wells of the elution plate for assay validity. Each result was multiplied by their associated dilution (calculated dilution factor assumes 1.67 ml of plasma per spot assayed) for their equivalent concentrations in nanograms per milliliter. This dilution factor may need to be adjusted in the future on the basis of assay concentration comparisons of DBS versus serum values for matched samples (*26*).

### Comparison of growth velocity to Cxm levels in mouse

DBS samples were obtained from 2-, 3-, 4-, 6-, 8-, 10-, and 12-week-old mice. After blood collection, mice were euthanized, and the lengths of tails and dissected femurs and tibias were measured with calipers.Femur and tibia measurements were averaged from both limbs. Individual growth rates were derived by the following formulas: change in length = (length measurement of individual) − (average length of all individuals at previous time point); growth velocity = change in length / elapsed time between measurements. Elution and measurement of DBS Cxm were performed according to procedures described above.

### Half-life testing

Twomale andthree female 25-week-oldFVB-8micewith 0 to1.5ng/ml baseline levels of endogenous Cxm were injected intravenously with 532 ng of mouse rNC1 into their tail veins. Blood was sampled from tail or saphenous veins at roughly 10, 30, 60, 120, and 240 min after injection. The Cxm concentration determined for the 10-min time point was set at 100%. Subsequent sampling and concentrations were plotted as a percentage of the initial value for eachmouse in the study.

### Statistical analysis

Across the mouse and human samples, CXM was plotted against age to show growth curves and with superimposed established growth velocity curves for comparison for humans. For tests of association of CXM with growth velocity, scatterplots and linear fit summary lines were generated, and Pearson’s correlation and statistical significance was calculated. A power series–fitted summary line was generated to summarize the nonlinear relationship of CXM to growth velocity in healthy children (Fig. 7). The criterion *P* value was set at *P* < 0.01 for all tests of significance. This study tested a small number of theoretically targeted relationships, so no adjustment was made of criterion *P* values for multiple comparisons. All statistical analysis was performed using GraphPad Prism 7 and Stata 14. Lower limit of quantitation calculations were performed using statistical equations published by Armbruster *et al.* (*42*).

## SUPPLEMENTARY MATERIALS

www.sciencetranslationalmedicine.org/cgi/content/full/9/419/eaan4669/DC1 Materials and Methods

Fig. S1. Fully tryptic fragments generated in mass spectrometry analysis.

Fig. S2. Dissociation of mouse rNC1 into dimers and monomers.

Fig. S3. Lower limit of quantitation.

Fig. S4. CXM stability testing.

Fig. S5. Relationship of stadiometer-based height velocities to CXM.

Fig. S6. Relationship between serum, plasma, and DBS concentrations.

Fig. S7. Half-life of CXM.

Table S1. Technical characterization of CXM assay.

Table S2. Diurnal variation data.

Table S3. Blood sample data.

## Citation

R. F. Coghlan, J. A. Oberdorf, S. Sienko, M. D. Aiona, B. A. Boston, K. J. Connelly, C. Bahney, J. LaRouche, S. M. Almubarak, D. T. Coleman, I. Girkontaite, K. von der Mark, G. P. Lunstrum, W. A. Horton, A degradation fragment of type X collagen is a real-time marker for bone growth velocity. *Sci. Transl. Med.* ***9***, eaan4669 (2017).

## Data and materials availability

Antibodies used in this study are available from W.A.H. under a material transfer agreement with Shriners Hospitals for Children.
