# Supplementary material for: A degradation fragment of type X collagen is a real-time marker for bone growth velocity
Source: Sci Transl Med. 2017 Dec 6;9(419):eaan4669. doi: 10.1126/scitranslmed.aan4669 (PMC6516194; doi:10.1126/scitranslmed.aan4669)
Supplement: Supplementary file 1 [file STM-09-eaan4669-s1.pdf]

Science Translational Medicine
AAAS

www.sciencetranslationalmedicine.org/cgi/content/full/9/419/eaan4669/DC1

Supplementary Materials for

A degradation fragment of type X collagen is a real-time marker for bone growth velocity

Ryan F. Coghlan, Jon A. Oberdorf, Susan Sienko, Michael D. Aiona, Bruce A. Boston, Kara J. Connelly, Chelsea Bahney, Jeremie LaRouche, Sarah M. Almubarak, Daniel T. Coleman, Irute Girkontaite, Klaus von der Mark, Gregory P. Lunstrum, William A. Horton*

*Corresponding author. Email: wah@shcc.org

Published 6 December 2017, *Sci. Transl. Med.* **9**, eaan4669 (2017)
DOI: 10.1126/scitranslmed.aan4669

This PDF file includes:

Materials and Methods
Fig. S1. Fully tryptic fragments generated in mass spectrometry analysis.
Fig. S2. Dissociation of mouse rNC1 into dimers and monomers.
Fig. S3. Lower limit of quantitation.
Fig. S4. CXM stability testing.
Fig. S5. Relationship of stadiometer-based height velocities to CXM.
Fig. S6. Relationship between serum, plasma, and DBS concentrations.
Fig. S7. Half-life of CXM.
Table S1. Technical characterization of CXM assay.
Table S2. Diurnal variation data.
Table S3. Blood sample data.

Materials and Methods

LLOQ calculations, experimental testing

Using calculations published by Armbruster et al (*42*), our lower limit of blank (LOB) for the CXM assay was determined to be 0.0722 ABS units at 450 nm. Lower limit of quantitation (LLOQ) testing (Fig. S3) was performed by diluting human rNC1 calibrator to concentrations of 7.5, 6.25, 4.5, and 3.13 pg/ml and running 16 separate replicates in a CXM ELISA. From this data we were able to calculate the theoretical limit of detection (LOD) as 0.0837 ABS units at 450 nm and the LLOQ as 0.1139 ABS units at 450 nm. This LLOQ value equates to 5.4 pg/ml.

CXM stability testing

For freeze/thaw analysis five separate serum samples from children in our study 1.6-12 years of age were thawed and assayed by CXM ELISA for an initial determination. Samples were then re-frozen at -20°C for 18 hours, thawed, and sampled again. This process was repeated for 5 freeze-thaw cycles. CXM concentrations for each subsequent freeze-thaw step were compared to the initial value and percent recovered plotted. (Fig. S4A).

For temperature stability analysis cord serum, serum, and plasma samples were thawed, aliquoted, and incubated at 4°C, 25°C, 37°C, or 50°C conditions for 18 hours. Samples were then assayed by CXM ELISA and the result for each temperature treatment was compared to their respective 4°C measurement (Fig. S4B).

DBS stability analysis utilized Whatman 903™ protein saver cards spotted with umbilical cord blood. Dried cards were placed in re-sealable bags with desiccant and stored for 8 days at -20°C, 4°C, 23°C (on bench), 23°C in envelope (on bench), 23°C (in variable sunlight, on windowsill),

at 37°C, at 37°C in a cell culture incubator (card placed in a petri dish instead of the re-sealable bag, no desiccant, in >95% humidity controlled, 5% CO_2 incubator), and at 55°C. After incubation 3.1mm punches were eluted and assayed by CXM ELISA and the resulting concentrations were compared as a percentage of the -20°C measurement (Fig. S4C).

Manufacture of SOMA1-Capture ELISA Plates

The SOMA1 reagent described proved effective at capturing both human and mouse markers, so the procedure below was used to generate plates for both assays.

STREPTAVIDIN: 100 µl/well of streptavidin (4 µg/ml in Coating Buffer) was added to each well of a 96 well 'High Bind' ELISA plate and incubated overnight at 4°C. Wash the next day with PBS (3 X 300 µl/well).

BLOCK 1: Plates were blocked for 1 hour by adding 300 µl/well of Blocking Buffer at RT and washed with PBS (3 X 300 µl/well).

SOMA1: 100 µl/well of biotinylated SOMA1 (3 pmoles/ml in SOMAmer Plating Buffer) was added to plates and incubated overnight at 4°C. Wash the next day with PBS (5 X 300 µl/well).

BLOCK 2: Plates were blocked for 10 minutes with 300 µl/well of Superblock at RT, emptied, and patted dry on paper towels to remove excess Superblock.

DRY: The plates were then dried in a desiccator at RT (until desiccator environment reached less than 10% humidity).

STORAGE: Plates were individually sealed in foil bags with desiccant pouches and stored at 4°C until use.

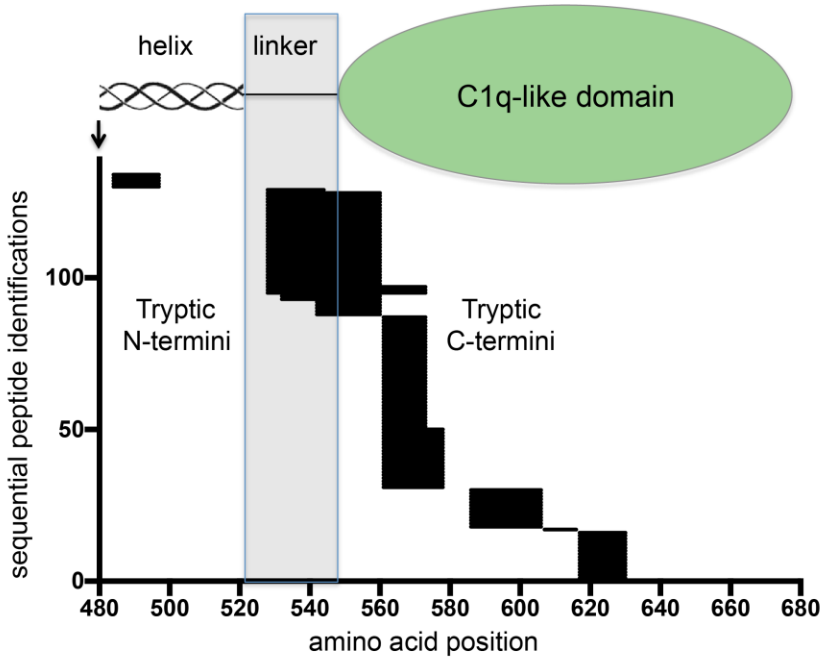

Fig. S1. Fully tryptic fragments generated in mass spectrometry analysis. Tryptic high-confidence peptide sequences are represented by stacked horizontal lines corresponding to their placement within the CXM marker. Proposed collagenase cut site (↓) corresponds to beginning of X-axis. Functional domains are diagrammed above graph with the linker region defined by a shaded box.

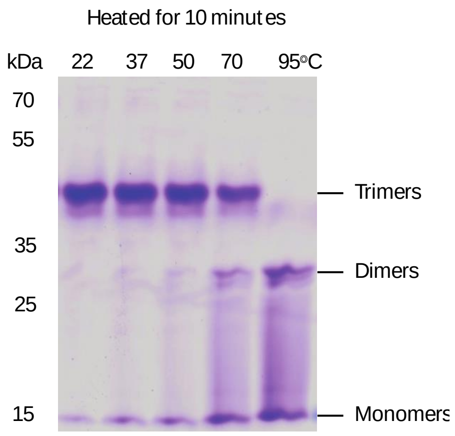

Fig. S2. **Dissociation of mouse rNC1 into dimers and monomers.** 3ug/lane of the recombinant NC1 region of mouse type X collagen was analyzed on SDS-PAGE after incubation in gel loading buffer for 10 minutes at the indicated temperatures. Protein was visualized with Coomassie stain.

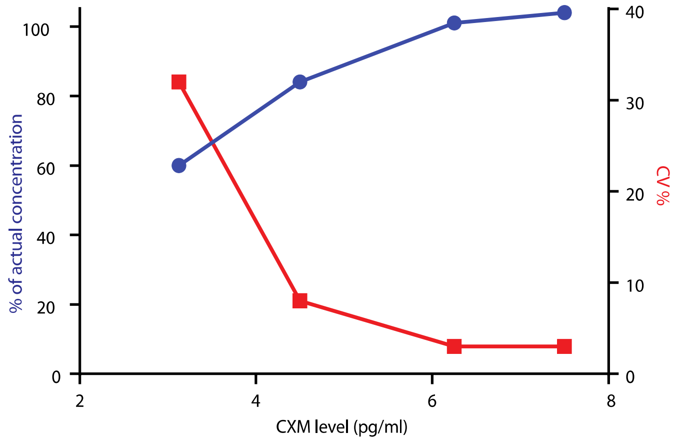

Fig. S3. Lower limit of quantitation. LLOQ testing was performed by diluting rNC1 calibrator to extremely low levels and calculating concentration CV% for each level (red). Concentrations determined for each sample were plotted as a percentage of their actual concentration (blue). The calculated LLOQ of 5.4 pg/ml is supported by this experimental data.

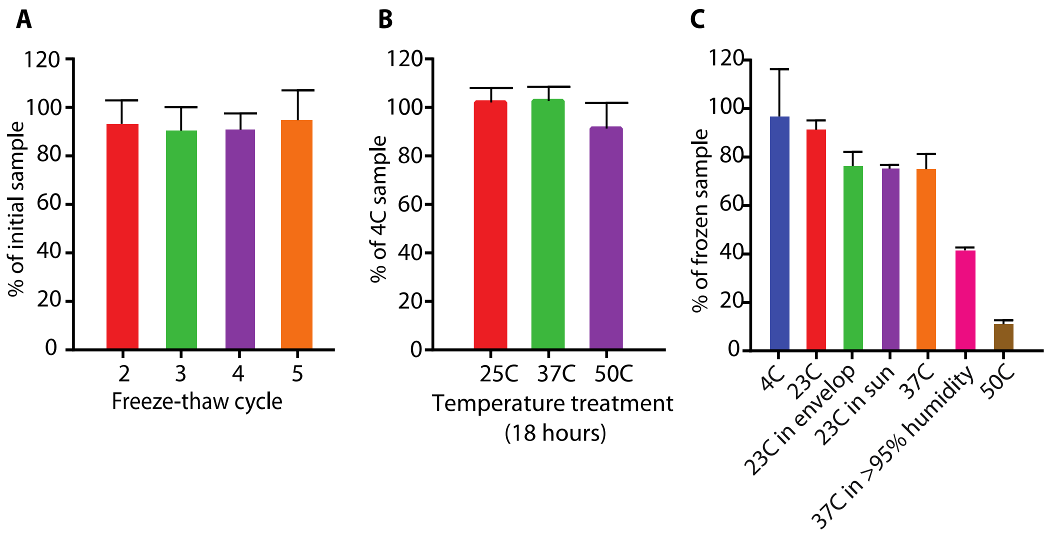

Fig. S4. **CXM stability testing.** CXM stability data showing variances from repeated freeze-thaw cycles, temperature stresses, and storage conditions. SD bars shown. (A) Percentage of recovery for serum samples cycled through 5 freeze/thaws with first freeze/thaw sample used as standard for comparison (n=5). (B) Cord serum, child serum, and child plasma temperature treatment concentrations compared as a percentage of their individual 4°C value but plotted together for statistical analysis (n=3). (C) DBS stability conditions are compared as a percentage of -20°C DBS sample (n=4, 8 day treatment).

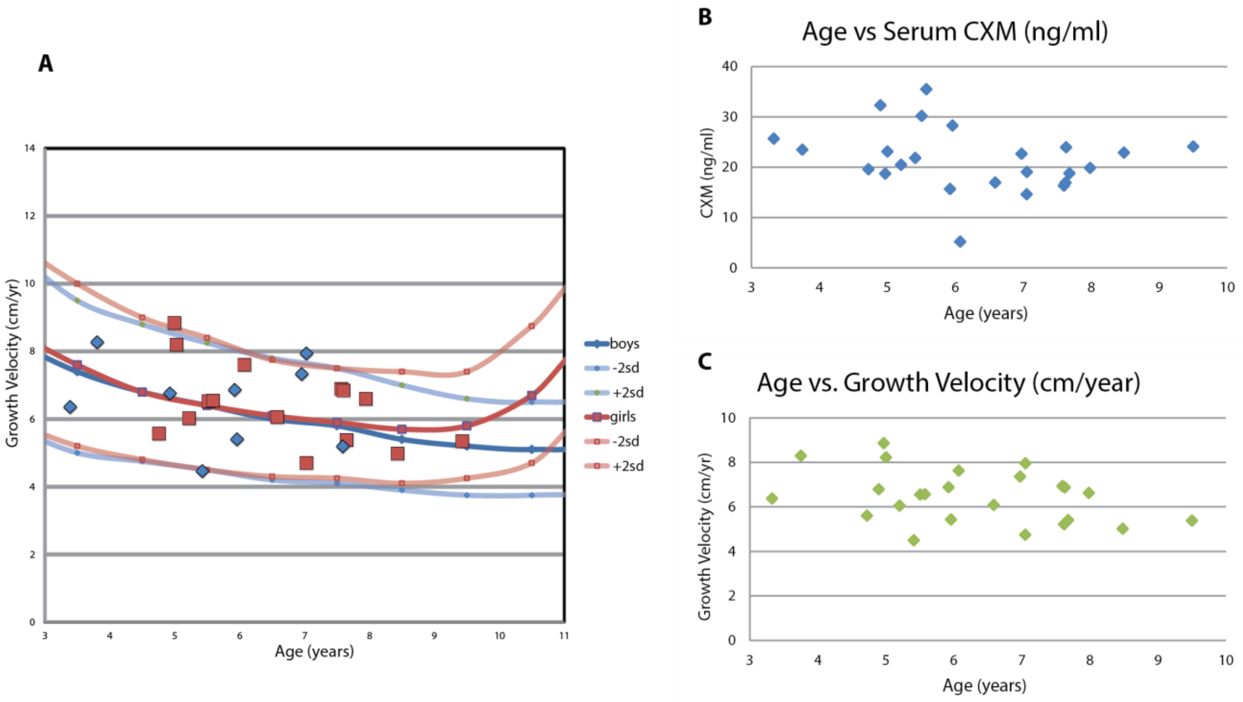

Fig. S5. Relationship of stadiometer-based height velocities to CXM. 23 subjects between ages 3.3 and 9.5 years whose data was used in the CXM vs observed height velocity analysis. A. Essentially all of our subjects fell within the normal ±2SD range when their measured growth velocities were plotted against established norms for age (*9*). B and C. Comparison of CXM values and observed height velocity to age, respectively. Both show a slight decline with age as expected. A visual inspection of the two plots suggests a greater dispersion for the CXM values (A) compared to observed height velocity (B).

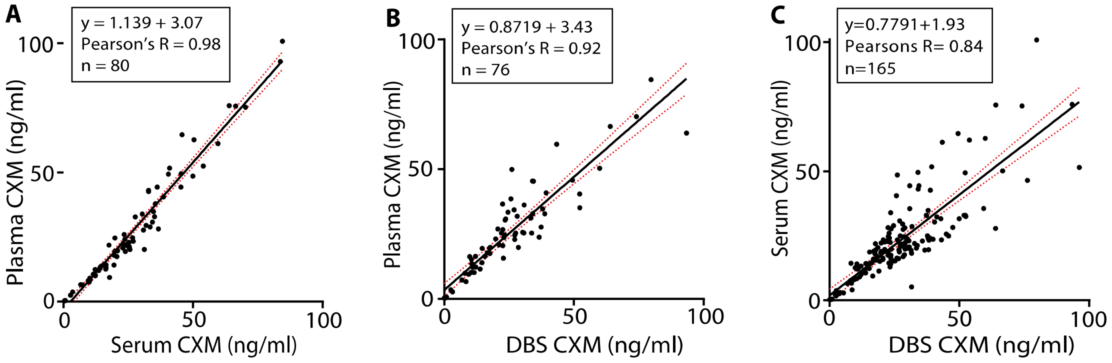

Fig. S6. **Relationship between serum, plasma, and DBS concentrations.** Serum, plasma, and DBS concentrations from paired samples are compared. The best-fit linear regression line is shown for each comparison in black, with 95% CI shown in red.

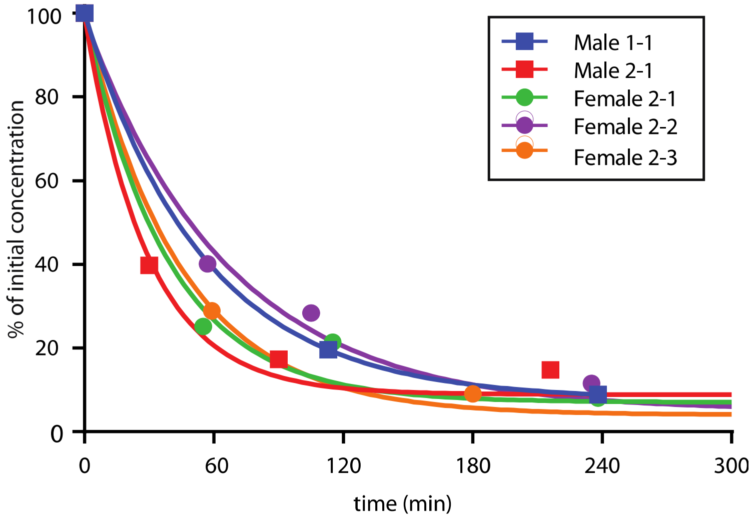

Fig. S7. **Half-life of Cxm.** Half-life of rNC1 was determined by injecting mouse rNC1 into 5 adult mice. Time zero corresponds to the initial blood sample 10 minute after injection. Best fit curves for each mouse were created in Prism using non-linear fit of one-phase decay.

Table S1. Technical characterization of CXM assay.

Technical Validation Step	Matrix / Analyte tested			
	**rNC1**	**Serum**	**Plasma**	**DBS**
Detection range	5.4-800 pg/ml	5.4-800 pg/ml	5.4-800 pg/ml	5.4-800 pg/ml
Lower limit of quantitation	5.4 pg/ml	N/A	N/A	N/A
Intra-assay variation (CV%)	4%	3%	3%	4%
Inter-assay variation (CV%)	8%	5%	3%	11%
Suggested dilution (children)	N/A	1:200	1:200	1x3.1mm punch in 300ul SD (1:178 dilution)
Suggested dilution (adults)	N/A	1:20	1:20	2x3.1mm punch in 240ul SD (1:72 dilution)
Matched samples as % of serum value	N/A	N/A	107%	125%
Analyte stability (4C/-20C)	99% (18 h)	96% (18 h)	101% (18 h)	97% (8 days)
Analyte stability (20C/-20C)	96% (18 h)	92% (18 h)	104% (18 h)	91% (8 days)
Analyte stability (37C/-20C)	101% (18 h)	102% (18 h)	97% (18 h)	75% (8 days)
Analyte stability (50C/-20C)	102% (18 h)	87% (18 h)	81% (18 h)	11% (8 days)

Abbreviations: h-hours. C: Degrees Celsius

Table S2. Diurnal variation data.

Multi-week diurnal samples

Sample ID	Sex	Age (years)	Week	Day	Hour	CXM (ng/ml)
107	F	2	1	1	9.75	39.81
107	F	2	1	1	17.33	35.63
107	F	2	1	1	19.25	34.63
107	F	2	1	2	9.95	46.83
107	F	2	1	2	16.25	34.62
107	F	2	1	2	19.25	33.24
107	F	2	1	2	21.87	35.60
107	F	2	1	3	10.17	44.13
107	F	2	1	3	18.65	33.09
107	F	2	1	3	20.53	22.32
107	F	2	1	3	22.75	31.98
107	F	2	2	1	9.83	39.92
107	F	2	2	1	17.00	28.89
107	F	2	2	1	20.50	33.89
107	F	2	2	2	9.87	45.93
107	F	2	2	2	13.83	41.53
107	F	2	2	2	17.50	36.64
107	F	2	3	1	9.67	41.21
107	F	2	3	1	14.25	27.51
107	F	2	3	1	20.42	16.18
107	F	2	3	2	9.42	35.16
107	F	2	3	2	13.42	29.09
107	F	2	3	2	20.50	23.30
107	F	2	3	3	13.83	25.62
107	F	2	3	3	17.67	20.96
107	F	2	3	3	19.92	25.33
108	F	4	1	1	10.75	23.61
108	F	4	1	1	15.50	25.46
108	F	4	1	1	20.50	18.07
108	F	4	1	2	9.50	24.82
108	F	4	1	2	13.00	22.30
108	F	4	1	2	19.00	22.09
108	F	4	1	3	9.00	24.78
108	F	4	1	3	12.75	23.29
108	F	4	1	3	20.75	22.04
108	F	4	2	1	11.00	25.43
108	F	4	2	1	17.00	24.17
108	F	4	2	1	19.75	24.10
108	F	4	2	2	11.00	37.00
108	F	4	2	2	14.17	28.21
108	F	4	2	2	19.75	22.38
108	F	4	2	3	11.00	31.85
108	F	4	2	3	14.00	28.62
108	F	4	2	3	18.00	24.86
108	F	4	3	1	9.00	23.56
108	F	4	3	1	14.00	14.28
108	F	4	3	1	22.00	16.66
108	F	4	3	2	10.50	28.44
108	F	4	3	2	13.50	21.67
108	F	4	3	2	20.75	20.08
108	F	4	3	3	11.00	24.64
108	F	4	3	3	15.00	18.61
108	F	4	3	3	21.83	18.44
116	F	11	1	1	9.58	31.78
116	F	11	1	1	17.08	36.29
116	F	11	1	1	23.33	45.12
116	F	11	1	2	9.67	56.89
116	F	11	1	2	19.25	34.23
116	F	11	1	2	21.60	40.24
116	F	11	1	3	8.65	42.20
116	F	11	1	3	16.83	45.95
116	F	11	1	3	22.67	44.88
116	F	11	2	1	13.00	41.52
116	F	11	2	1	21.05	27.96
116	F	11	2	1	23.75	22.97
116	F	11	2	2	9.85	33.42
116	F	11	2	2	13.20	42.71
116	F	11	2	2	19.25	31.09
116	F	11	2	2	20.98	28.79
116	F	11	2	3	7.50	23.14
116	F	11	2	3	18.77	32.59
116	F	11	2	3	22.83	26.01
116	F	11	3	1	8.52	36.14
116	F	11	3	1	19.12	29.63
116	F	11	3	1	22.27	28.54
116	F	11	3	2	8.38	36.83
116	F	11	3	3	8.82	23.42
116	F	11	3	3	19.35	33.34
116	F	11	3	3	21.85	27.98
116	F	11	3	4	11.68	37.59
116	F	11	3	4	22.25	26.77

Single week diurnal samples

Sample ID	Sex	Age (years)	Day	Hour	CXM (ng/ml)
101	M	4	1	13.75	20.42
101	M	4	2	13.83	25.30
101	M	4	2	17.83	22.49
101	M	4	2	20.25	20.86
101	M	4	3	6.93	34.78
101	M	4	3	10.48	25.68
101	M	4	4	17.25	20.46
101	M	4	4	20.25	19.40
101	M	4	5	7.25	26.81
103	F	11	1	10.00	20.68
103	F	11	1	18.87	16.80
103	F	11	1	22.62	12.93
103	F	11	2	10.03	25.13
103	F	11	2	21.60	18.66
103	F	11	2	22.50	16.90
103	F	11	3	10.02	20.51
103	F	11	3	15.22	18.10
103	F	11	3	22.00	19.06
105	M	7	1	10.08	22.81
105	M	7	1	19.50	12.99
105	M	7	1	23.00	17.64
105	M	7	2	10.00	10.04
105	M	7	2	18.00	11.89
105	M	7	3	10.00	14.50
105	M	7	3	18.50	15.77
105	M	7	3	23.00	16.63
113	M	14	1	10.77	30.93
113	M	14	1	19.67	24.57
113	M	14	2	11.00	29.43
113	M	14	2	11.00	28.28
113	M	14	2	19.00	28.89
113	M	14	3	11.00	31.18
113	M	14	3	13.00	26.42
113	M	14	4	10.50	38.95
113	M	14	4	18.50	29.73
113	M	14	4	22.50	31.23
117	F	7	1	8.00	17.53
117	F	7	1	18.50	15.54
117	F	7	1	20.50	14.57
117	F	7	2	9.25	15.87
117	F	7	2	17.50	15.81
117	F	7	2	20.50	11.29
117	F	7	3	6.00	13.70
117	F	7	3	18.50	12.25
117	F	7	3	21.00	10.65
115	F	5	1	8.32	11.81
115	F	5	1	17.42	10.17
115	F	5	1	21.63	9.28
115	F	5	2	7.58	17.47
115	F	5	2	17.72	9.64
115	F	5	2	21.80	7.66
115	F	5	3	17.38	16.14
115	F	5	3	22.23	9.18
118	F	13	1	6.83	18.53
118	F	13	1	17.75	12.34
118	F	13	1	21.82	11.70
118	F	13	2	8.88	14.82
118	F	13	2	20.60	12.90
118	F	13	2	22.62	12.85
118	F	13	3	7.93	18.36
118	F	13	3	21.25	13.00
106	M	10	1	8.80	14.48
106	M	10	1	18.40	13.36
106	M	10	1	21.00	12.06
106	M	10	2	9.42	15.32
106	M	10	2	18.08	11.87
106	M	10	2	20.58	9.10
106	M	10	3	7.17	13.92
106	M	10	3	18.50	10.83
106	M	10	3	20.50	10.31
119	M	11	1	7.78	30.30
119	M	11	1	19.93	21.16
119	M	11	1	20.73	15.90
119	M	11	2	8.40	28.26
119	M	11	2	18.52	17.30
119	M	11	2	19.77	18.71
119	M	11	3	10.02	26.07
119	M	11	3	17.33	15.67
119	M	11	3	21.07	15.22

Analysis of all Diurnal samples grouped by Sample ID

Sample ID	Sex	Age (years)	Average CXM before 2pm (ng/ml)	n (before 2pm samples)	Average CXM after 2pm (ng/ml)	n (after 2pm samples)	Average morning CXM/ Average afternoon CXM percentage
107	F	2	38.92	10	29.61	16	131.43%
108	F	4	25.31	14	21.94	13	115.37%
116	F	11	36.88	11	33.08	17	111.47%
101	M	4	26.60	5	20.80	4	127.85%
103	F	11	22.11	3	17.07	6	129.47%
105	M	7	15.78	3	14.98	5	105.33%
113	M	14	30.86	6	28.61	4	107.89%
117	F	7	15.70	3	13.35	6	117.60%
115	F	5	14.64	2	10.35	6	141.49%
118	F	13	17.24	3	12.56	5	137.26%
106	M	10	14.57	3	11.25	6	129.49%
119	M	11	28.21	3	17.33	6	162.81%
					Overall average:		126.45%

Table S3. Blood sample data.

ID no	Age at draw (years)	Sex	Height (cm)	Growth Velocity (cm/year)	Serum CXM (ng/ml)	Serum CXM CV%	Plasma CXM (ng/ml)	Plasma CXM CV%	DBS CXM (ng/ml)	DBS CXM CV%
NO65	0.06	M	56.5		92.86	4%	83.73	3%		
N066	0.17	M	61.0		52.46	6%	53.96	6%		
N067	0.67	F	69.4		75.24	7%	70.28	3%	74.10	5%
NO68	9.08	M	131.0		20.65	0%	25.49	2%	30.73	4%
NO69	8.92	M	136.9		19.43	2%	23.85	1%	36.63	1%
NO70	0.67	M	69.0		37.92	5%	35.44	3%		
NO71	5.50	M	114.9		17.47	0%	20.58	2%	22.61	8%
NO72	0.67	M	77.0		64.57	1%	45.81	1%	49.51	1%
NO73	0.92	F	77.0		62.69	6%	50.40	3%	59.98	1%
NO74	0.75	F	73.0		42.60	2%	32.89	5%	38.81	3%
NO75	12.00	M	155.7		20.20	4%	31.20	1%	33.45	2%
NO76	4.42	F	107.2		32.87	3%	35.16	3%	52.29	3%
NO77	1.58	M	84.2		75.65	5%	66.51	1%	64.01	1%
NO78	0.17	F	55.0		75.81	7%	63.95	3%	93.41	6%
NO79	1.58	M	82.0		32.81	4%	27.70	0%	37.39	6%
NO80	12.33	F	162.9		18.79	2%			26.86	4%
NO81	13.42	M	152.2		23.01	2%	25.36	2%	22.06	9%
NO82	0.67	F	71.0		49.35	1%	40.46	1%	52.20	5%
NO83	2.83	F	95.7		44.32	1%	36.20	3%	30.83	18%
NO84	4.67	M	102.9		24.62	2%	19.84	1%	28.60	3%
NO85	13.75	F	159.9		14.10	3%	15.26	2%	10.48	8%
NO86	0.13	M	55.0		100.78	5%	84.51	2%	79.76	3%
NO87	12.33	F	152.4		21.26	1%	25.40	2%	33.77	7%
NO88	7.58	M	131.1		19.68	1%	20.86	2%	21.16	4%
NO89	7.42	M	132.4		12.87	2%	13.97	3%	14.59	2%
NO90	9.00	M	132.4		12.12	2%	13.64	3%	10.62	1%
NO91	7.00	M	121.0		13.22	5%	16.42	7%	12.05	4%
NO92	3.17	F	93.9		51.69	1%	40.93	4%	39.33	7%
NO93	1.83	M	80.5		24.14	1%	23.66	4%	23.03	1%
NO94	1.25	F	70.0		43.07	4%	32.83	9%	34.08	1%
NO95	0.75	M	73.5		61.19	5%	59.63	1%	43.40	7%
NO96	5.50	M	104.7		14.16	2%	19.50	5%	14.68	4%
NO97	13.33	F	159.8		14.28	2%	16.42	1%	15.69	3%
NO98	5.50	M	112.3		9.41	3%	17.75	1%	16.56	0%
NO99	6.33	M	110.8		14.43	2%	15.67	1%	22.86	5%
N100	14.08	F	164.6		3.79	0%	3.52	2%	2.57	5%
N101	12.83	M	167.1		10.65	6%	11.55	3%	13.84	6%
N102	15.58	M	176.5		6.60	5%	6.66	0%	8.44	1%
N103	11.33	M	148.0		21.64	5%	24.59	2%	27.24	1%
N104	12.00	F	160.8		10.07	1%	12.26	0%	12.22	1%
N105	12.00	F	156.0		12.60	2%	16.26	7%	9.83	4%
N106	8.33	F	127.2		28.16	1%	36.61	2%	22.53	2%
N107	0.50	M	67.0		62.04	2%			53.89	3%
SG2001	28.50	F	166.5		0.37	5%	0.44	2%	0.48	10%
SG2002	23.08	F	166.1		0.24	9%	0.30	5%	0.00	
SG2003	29.83	M	177.6		0.11	17%	0.16	9%	0.00	
SG2004	26.67	F	165.2		0.44	0%	0.51	3%	0.58	7%
SG2005	29.00	F	147.0		0.29	8%	0.45	2%	0.23	9%
SG2006	28.00	M	196.6		0.20	0%	0.28	2%	0.00	
SG2007	28.42	M	186.4		0.31	1%	0.24	8%	0.13	8%
SG2008	31.00	M	174.7		0.32	3%	0.29	5%	0.70	
SG2009	27.33	F	173.3		0.34	7%	0.25	3%	0.32	5%
SG2010	20.08	F	168.3		0.51	1%	0.69	6%	0.92	8%
NG1001c	1.31	F	78.5	11.080	20.91	4%			28.45	1%
NG1002a	4.91	M	106.0		29.43	12%				
NG1002b	5.45	M			19.62	3%			38.09	0%
NG1002c	5.96	M	111.7	5.432	28.25	2%			47.48	3%
NG1003a	4.50	F	110.0		25.11	5%				
NG1003b	5.00	F	114.1	8.223	23.08	1%			30.53	3%
NG1003c	5.51	F	117.4	6.546	30.14	4%				
NG1004	3.70	M	102.0		16.17	2%			26.55	2%
NG1005a	2.08	F	83.0		28.31	0%				
NG1005b	2.65	F	85.6	4.562	27.49	2%			28.34	6%
NG1006a	0.25	M	61.1		27.89	1%			63.96	2%
NG1006b	0.70	M	70.7	21.764	24.13	5%			38.62	7%
NG1007a	0.90	F	74.6		46.47	9%			76.22	10%
NG1007b	1.46	F	82.5	14.066	35.59	5%			59.35	2%
NG1007c	2.03	F	88.2	10.002	21.99	2%			32.69	5%
NG1008	0.45	M	65.3		32.48	0%			52.44	5%
NG1009	0.92	F	73.5		24.94	2%			45.82	3%
NG1009c	2.57	F	90.0	10.004	18.91	8%			21.91	7%
NG1010a	2.98	M	99.1		23.67	5%			39.83	9%
NG1010b	3.33	M	101.3	6.373	25.66	4%			44.94	1%
NG1010c	3.75	M	104.8	8.295	23.44	8%			43.06	18%
NG1011a	4.47	M	101.0		23.27	6%			38.27	1%
NG1011b	4.90	M	103.9	6.785	32.27	8%			41.30	2%
NG1011c	5.41	M	106.2	4.489	21.80	2%			27.72	2%
NG1012a	0.18	F	50.0		51.47	2%			96.14	0%
NG1012b	0.53	F	62.0	34.762	50.09	3%			66.65	2%
NG1013a	4.21	F	102.2		17.67	0%			28.83	3%
NG1013b	4.72	F	105.1	5.601	19.57	1%			26.66	2%
NG1013c	5.20	F	108.0	6.049	20.43	1%			18.59	2%
NG1014a	1.34	M	77.5		23.80	7%			29.92	7%
NG1014b	1.66	M	82.7	16.222	22.40	3%			29.14	6%
NG1014c	2.33	M	89.4	9.982	24.96	3%			27.20	7%
NG1015	0.55	F	70.0		31.59	1%			49.95	1%
NG1016a	0.97	F	73.0		25.03	4%			37.65	12%
NG1016b	1.32	F	77.9	14.194	32.98	9%			37.23	2%
NG1016c	2.29	F	87.0	9.304	31.58	6%			42.86	0%
NG1017a	3.66	F	99.5		28.59	1%			31.76	12%
NG1017b	4.97	F	111.1	8.858	18.70	3%			15.43	4%
NG2001a	7.21	F	127.3		26.20	5%				
NG2001b	7.63	F	130.2	6.873	23.92	2%				
NG2002	15.39	M	154.9		32.04	4%				
NG2003	5.59	F	107.9		25.52	1%				
NG2004a	5.48	M	111.1		11.95	4%				
NG2004b	5.92	M	123.8	6.878	15.66	0%			16.91	2%
NG2004c	7.05	M	132.8	7.954	19.02	1%			16.96	3%
NG2005	8.75	M	117.0		14.85	1%				
NG2006	5.25	M	114.0		25.99	4%				
NG2006b	6.98	M	126.7	7.358	22.63	1%			21.22	4%
NG2007a	8.89	F	137.5		25.07	2%				0%
NG2007b	9.50	F	140.8	5.377	24.06	5%			37.98	2%
NG2007c	10.02	F	143.5	5.214	17.76	3%				0%
NG2008a	6.66	F	113.9		24.35	5%			29.31	0%
NG2008b	7.99	F	122.7	6.623	19.84	1%			23.77	1%
NG2008c	8.48	F	125.2	5.014	22.87	0%			22.48	0%
NG2009a	9.95	F	156.7		32.25	5%				
NG2009b	10.42	F	161.4	9.803	32.93	2%			51.79	9%
NG2009c	10.90	F	165.7	9.072	30.31	2%			40.42	5%
NG2010a	15.03	F	154.0		3.07	3%			3.95	1%
NG2010b	15.49	F	154.2	0.435	2.18	7%			2.22	3%
NG2010c	16.01	F	154.3	0.192	1.73	1%			2.50	3%
NG2011	8.81	F	134.5		19.36	1%			31.37	2%
NG2012a	7.09	M	117.3		11.34	3%			18.99	4%
NG2012b	7.62	M	120.1	5.214	16.91	0%			28.33	0%
NG2012c	8.12	M			14.27	4%			13.99	5%
NG2013a	13.10	M	156.9		15.59	0%			17.72	3%
NG2013b	13.53	M	158.6	3.978	17.45	2%			23.66	1%
NG2014	11.23	F	159.7		22.03	1%			27.78	4%
NG2015	5.25	M	111.8		15.24	1%			24.51	6%
NG2016a	5.90	F	116.4		15.83	1%			20.49	1%
NG2016b	6.59	F	120.6	6.083	16.92	4%			18.42	5%
NG2016c	7.60	F	127.6	6.924	16.32	4%			20.50	3%
NG2017a	6.42	F	116.1		18.26	2%			31.57	1%
NG2017b	7.05	F	119.1	4.740	14.59	3%			16.33	4%
NG2018a	5.06	F	109.4		22.98	3%			35.76	1%
NG2018b	5.58	F	112.8	6.566	35.48	1%			30.85	1%
NG2018c	6.07	F	116.6	7.621	5.19	1%			31.56	1%
NG2019a	12.61	F	150.9		4.06	0%			8.65	2%
NG2019b	13.10	F	151.0	0.201	2.86	9%			5.36	2%
NG2019c	13.74	F			2.60	5%			3.72	9%
NG2020	8.51	M	138.1		12.94	3%			23.26	2%
NG2021a	9.62	F	129.0		16.41	1%			25.93	2%
NG2021b	10.31	F	132.3	4.780	18.91	7%			19.90	1%
NG2021c	11.02	F	135.0	3.805	15.11	2%			19.37	1%
NG2022a	7.16	F	116.0		15.23	0%			25.18	2%
NG2022b	7.68	F	118.8	5.407	18.78	0%			22.52	5%
NG2023	8.35	F	130.7		19.83	1%			29.09	2%
S1002a	0.17	M			49.52	0%	45.43	4%	34.06	5%
S1002c	1.00	M			19.50	1%	19.88	6%	17.75	0%
S1004a	1.83	M			33.91	1%	30.40	3%	23.51	1%
S1004b	2.34	M			23.41	1%	26.94	1%	20.69	4%
S1004c	3.02	M			21.90	3%	17.53	0%	17.60	2%
S1005	5.17	F			30.80	3%	34.32	3%	26.99	0%
S1006	5.33	M			29.48	0%	31.88	4%	28.21	0%
S1007	0.33	M			40.50	5%	38.57	5%	25.75	0%
S1007c	2.09	M			34.86	4%	34.74	4%	37.99	2%
S1008a	0.58	M			48.57	1%	49.95	0%	26.07	15%
S1008b	1.09	M			44.32	1%	45.36	1%	34.42	0%
S1008c	1.59	M			28.89	1%	33.42	6%	24.62	1%
S1009a	0.58	M			27.72	7%	30.81	2%	23.71	1%
S1009b	1.17	M			24.71	3%	26.72	1%	22.58	5%
S2001a	12.67	M			18.02	1%	19.58	3%	17.50	0%
S2001b	13.37	M			20.83	1%	22.94	4%	27.24	2%
S2001c	13.68	M			22.43	5%	24.66	2%	23.33	1%
S2002a	14.25	M			6.45	5%	7.27	2%	6.90	6%
S2002c	15.31	M			2.51	10%	2.69	1%	3.23	2%
S2003a	7.00	F			8.15	0%	9.53	1%	9.44	2%
S2003b	7.60	F			7.57	3%	9.90	3%	8.00	3%
S2003c	8.06	F			9.12	3%	10.15	1%	11.52	2%
S2004	9.50	M			8.43	0%	10.28	2%	10.42	3%
S2005	11.58	F			12.43	3%	12.21	2%	11.00	7%
S2006	6.92	F			26.00	2%	23.46	1%	22.91	0%
S2007	10.92	F			22.22	2%	23.14	2%	19.85	2%
S2008	13.42	F			13.55	7%	14.38	2%		
S2009a	6.50	M			20.18	9%	25.68	1%	30.25	2%
S2009b	7.04	M			20.89	0%	25.35	4%	27.59	2%
G1001c	5.49	M			12.67	1%			10.92	1%
G1002c	3.77	M			20.72	6%			29.40	2%
G1003a	1.81	F			23.47	1%			26.06	4%
G1003b	3.31	F			28.06	3%			28.98	1%
G1004a	4.41	M			16.19	0%			23.64	2%
G2007c	14.55	M			18.46	2%			19.68	2%
G2008c	18.53	M			8.04	1%			9.45	3%
G2010b	7.07	F			10.93	6%			12.72	0%
G2010c	7.61	F			11.80	1%			13.44	2%
G2012a	5.27	F			14.37	1%			8.21	1%
G2013a	10.82	M			14.04	1%			23.53	3%
G2014a	8.62	M			12.30	0%			15.22	0%

Data in blue used for matched DBS, plasma, and serum comparisons only (fig. S5).
